# Pink lotus flower (*Nelumbo nucifera*) oil extract alleviates imiquimod-induced psoriasis-like dermatitis

**DOI:** 10.3892/br.2026.2163

**Published:** 2026-06-03

**Authors:** Kamonwan Jongsomchai, Somyoth Sridurongrit, Passanesh Sukphopetch, Tawut Rudtanatip, Tichanon Promsrisuk, Laorrat Phuapittayalert, Sataporn Jamsuwan, Teera Chanmanee, Sarinthorn Thummayot, Sitthisak Thongrong, Amnart Onsa-Ard, Arnon Pudgerd

**Affiliations:** 1 Division of Anatomy, School of Medical Sciences, University of Phayao, Phayao 56000, Thailand; 2 Department of Anatomy, Faculty of Science, Mahidol University, Bangkok 10400, Thailand; 3 Department of Microbiology and Immunology, Faculty of Tropical Medicine, Mahidol University, Bangkok 10400, Thailand; 4 Electron Microscopy Unit, Department of Anatomy, Faculty of Medicine, Khon Kaen University, Khon Kaen 40002, Thailand; 5 Division of Physiology, School of Medical Sciences, University of Phayao, Phayao 56000, Thailand; 6 Division of Biochemistry, School of Medical Sciences, University of Phayao 56000, Thailand

**Keywords:** pink lotus oil, imiquimod, psoriasis, dermatitis, parakeratosis

## Abstract

Pink lotus (*Nelumbo nucifera* Gaertn.) contains bioactive flavonoids and alkaloids that exert anti-inflammatory and antioxidant activities, and inhibit TNF-α, IL-1β, IL-6 and NF-κB signaling pathways implicated in the pathogenesis of psoriasis. However, the effects of pink lotus flower oil (PLO) on psoriasis-like skin inflammation remain unexplored. Therefore, the present study aimed to evaluate the effects of PLO treatment on imiquimod (IMQ)-induced psoriasis-like skin inflammation in a mouse model. Briefly, BALB/c mice with IMQ-induced psoriasis were administered PLO (100 and 200 mg/kg), and the psoriasis area and severity index were measured. Histopathology, proinflammatory cytokine levels, antioxidant gene expression, oxidative stress and antioxidant marker levels, and immunoreactivity were also assessed. PLO treatment markedly reduced erythema, scaling and epidermal thickening, and suppressed keratinocyte proliferation, as evidenced by decreased proliferating cell nuclear antigen immunoreactivity, and downregulated mRNA levels of keratin-encoding genes *keratin* 6 (*K6*), *K16* and *K17*. Moreover, PLO significantly downregulated the levels of proinflammatory cytokines, including TNF-α, IL-17A, IL-23 and *IL-6*, and reduced the infiltration of CD4^+^ and mast cells. Mechanistically, PLO treatment decreased phosphorylated (p)-JAK2, p-JAK3 and p-STAT3 immunoreactivity by inhibiting JAK2/JAK3/STAT3 signaling. Oxidative stress was attenuated, as evidenced by reduced malondialdehyde levels, and increased nuclear factor erythroid 2-related factor 2, Cu/Zn-superoxide dismutase (*Cu/Zn-SOD*), *Mn-SOD*, catalase and glutathione peroxidase mRNA expression levels. PLO treatment also mitigated IMQ-induced splenomegaly, and suppressed *IL-1b and IL-6* expression in the spleen, and reduced *IL-6* expression in the axillary lymph nodes. In conclusion, PLO treatment ameliorated IMQ-induced psoriasis by enhancing antioxidant defenses, and by inhibiting JAK2 and JAK3/STAT3 signaling, supporting its potential as a therapeutic candidate for psoriasis.

## Introduction

Psoriasis is a chronic, immune-mediated inflammatory skin disorder characterized by well-demarcated erythematous plaques with silvery scales, predominantly affecting the scalp, elbows, knees and lower back. Histopathologically, psoriatic lesions are marked by epidermal hyperplasia (acanthosis), parakeratosis, elongated rete ridges and dense dermal infiltration of immune cells (1-3). Psoriasis markedly impairs quality of life and is associated with numerous comorbidities, including metabolic syndrome, cardiovascular disease and psoriatic arthritis (4,5).

Psoriasis is a multifactorial disease involving a complex interplay among keratinocytes, immune cells and proinflammatory mediators. Dysregulated activation of the IL-23/IL-17 axis promotes the differentiation and activation of T helper (Th)17 cells, leading to overproduction of IL-17A, IL-23, TNF-α, IL-1β and IL-6 (6,7). These cytokines activate downstream signaling pathways, such as NF-κB, JAK/STAT3 and MAPK, which amplify inflammation and keratinocyte hyperproliferation (8). STAT3 serves a pivotal role in psoriatic epidermis by modulating the expression of genes associated with proliferation and immune responses, including *TNF-α, IL-1b, S100A8* and *S100A9* (9).

Oxidative stress contributes to psoriasis; overproduction of reactive oxygen species in skin lesions promotes lipid peroxidation, DNA damage and aberrant activation of redox-sensitive transcription factors, including NF-κB (10). The nuclear factor erythroid 2-related factor 2 (Nrf2) pathway, a major cellular defense mechanism against oxidative stress, orchestrates the expression of antioxidant enzymes, such as superoxide dismutase (SOD), catalase (CAT) and glutathione peroxidase (GSH-Px) (11). Impairment of Nrf2 signaling has been implicated in the pathogenesis of psoriasis, whereas its activation attenuates cutaneous inflammation and restores redox homeostasis (12). Moreover, Nrf2 regulates keratinocyte differentiation by modulating the expression of structural proteins such as filaggrin (FLG), loricrin (LOR) and involucrin, which are key components of the skin barrier and are often reduced in psoriasis (13). Dysregulation of these proteins compromises epidermal barrier function, facilitating immune cell infiltration and sustaining inflammation (10).

Pink lotus (*Nelumbo nucifera*) is an aquatic plant commonly cultivated in Asia owing to its edible and medicinal value. Its flower contains various natural compounds, including flavonoids and alkaloids, which possess antioxidant and anti-inflammatory properties (14). Pink lotus flower oil (PLO) extract has several pharmacological benefits; it alleviates neurodegeneration and promotes neural regeneration in mouse models of neurological injury (15,16). In addition, PLO increases the levels of SOD, CAT and reduced glutathione (GSH), which protect cells against oxidative stress-induced damage (17). Moreover, PLO has been shown to ameliorate hepatic inflammation in a mouse model of lipopolysaccharide-induced liver injury (18). However, the anti-inflammatory effects of PLO in other immunological diseases remain unclear. Considering that PLO suppresses the production of crucial mediators of psoriatic inflammation, such as TNF-α, IL-6, IL-1β and NF-κB (19), it was hypothesized that PLO may decrease skin inflammation and alleviate psoriasis. Therefore, the present study aimed to evaluate the effect of PLO in a mouse model of imiquimod (IMQ)-induced psoriasis-like skin inflammation.

## Materials and methods

### PLO extract

The PLO used in the present study was obtained from Tropicalife Co., Ltd. (pink lotus absolute; product code: ESA-OPL0010). The essential oil used in the present study was a commercially available pink lotus absolute extract obtained from the manufacturer. According to the manufacturer, the extract was prepared from whole lotus flowers using an absolute extraction process with hexane. The chemical composition of PLO was characterized using gas chromatography-mass spectrometry (GC-MS) in a previous study (16), which identified palmitic acid ethyl ester (25.12%), linoleic acid ethyl ester (18.17%) and methyl 8,11,14-heptadecatrienoate (10.45%) as the predominant constituents. Additional components detected in lower proportions included heneicosane (7.26%), methyl 2-methylhexadecanoate (7.09%), palmitic acid methyl ester (2.79%), hexadecane (2.78%) and (6Z,9E)-heptadeca-6,9-diene (2.62%) (16).

### Mouse model of IMQ-induced psoriasis and treatment

A total of 80 male BALB/c mice (age, 6-8 weeks; body weight, 26.4±1.26 g) were purchased from Nomura Siam International Co., Ltd. The mice were housed under specific pathogen-free conditions with control humidity (50-70%), temperature (25±2˚C) and a 12/12-h light/dark cycle, and with *ad libitum* access to water and food. The mice were randomly divided into the following eight groups (n=10/group): i) Control group, mice were intraesophageally administered normal saline (NS), followed by topical application of petroleum cream; ii) methotrexate (MTX) control group, mice were intraesophageally administered 1.0 mg/kg MTX (cat. no. A6770; Sigma-Aldrich; Merck KGaA) (20), followed by topical application of petroleum cream. MTX, an immunosuppressive and anti-psoriatic agent, was used as positive control because it is commonly prescribed as a first-line treatment for moderate-to-severe plaque psoriasis (20). iii) PLO-100 control group, mice were intraesophageally administered 100 mg/kg PLO, followed by topical application of petroleum cream. iv) PLO-200 control group, mice were intraesophageally administered 200 mg/kg PLO, followed by topical application of petroleum cream; v) IMQ + NS group, mice were intraesophageally administered NS, followed by topical application of IMQ cream. vi) IMQ + MTX group, mice were intraesophageally administered 1.0 mg/kg MTX, followed by topical application of IMQ cream. vii) IMQ + PLO-100 group, mice were intraesophageally administered 100 mg/kg PLO, followed by topical application of IMQ cream; and viii) IMQ + PLO-200 group, mice were intraesophageally administered 200 mg/kg PLO, followed by topical application of IMQ cream. Psoriasis-like dermatitis was induced by applying 62.5 mg 5% IMQ cream (Aldara^™^; Ensign Laboratories Pty Ltd.) daily to the shaved dorsal skin for 7 consecutive days. The PLO dosage was based on a previous study (15). The same amount of petroleum jelly (Vaseline^®^; Unilever) was applied to the shaved backs of the control mice (in groups i-iv).

The mice were weighed every other day. The severity of cutaneous inflammation was scored 24 h after IMQ application using the psoriasis area and severity index (PASI), including erythema, scaling and thickening. Each parameter was scored independently on a scale of 0-4 (0, none; 1, mild; 2, moderate; 3, marked; 4, severe). The cumulative score, which combined erythema, scaling and thickening scores, was used to evaluate the severity of skin inflammation. On day 8 of the experiment, the mice were euthanized by overexposure to 5% isoflurane delivered in oxygen using a veterinary anesthesia machine. After confirmation of deep anesthesia, the mice were maintained under 5% isoflurane during the thoracic procedure to ensure the absence of pain perception. Blood collection was then performed via cardiac puncture, which resulted in euthanasia of the animals. Isoflurane administration was subsequently discontinued, and the remaining tissue samples were collected thereafter. The major organs, including the lesional skin, heart, axillary lymph nodes, spleen, kidney, liver, lung, and small and large intestines, were collected for further analysis. The present study was approved by the Institutional Animal Care and Use Committee of the University of Phayao (approval no. 1-016-67; Phayao, Thailand).

### Sample preparation and cytokine detection. Blood serum

After euthanasia of the mice, the thoracic cavity was opened and blood samples were collected by cardiac puncture. The blood samples were allowed to clot at room temperature for 30 min and then centrifuged at 2,500 x g at 4˚C for 15 min. The supernatant (serum) was then collected and stored at -20˚C in preparation for ELISA.

*Skin tissue.* The dorsal skin of the control and experimental mice was collected, cut into 0.1-cm^3^ tissue sections and then added to 1 ml NS. The tissues were placed on ice, homogenized and centrifuged at 2,500 x g for 15 min at 4˚C. The supernatant was collected and stored at -20˚C for ELISA.

*Cytokine detection by ELISA.* The levels of proinflammatory cytokines in the dorsal skin and serum samples were determined using ELISA kits in accordance with the manufacturer's instructions. These cytokines included TNF-α (ELISA MAX™ Deluxe Set Mouse TNF-α Kit; cat. no. 430904), IL-1β (ELISA MAX Deluxe Set Mouse IL-1β Kit; cat. no. 432604), IL-10 (ELISA MAX Deluxe Set Mouse IL-10 Kit; cat. no. 431414), IL-17A (ELISA MAX Deluxe Set Mouse IL-17A Kit; cat. no. 432504) and IL-23 (ELISA MAX Deluxe Set Mouse IL-23 Kit; cat. no. 433704) (all from Biolegend, Inc.). Absorbance was measured at 450 nm on a spectrophotometer (VersaMax^™^ Microplate Reader; Molecular Devices, LLC) and the data were analyzed using SoftMax Pro software version 6 (Molecular Devices, LLC).

### Protein extraction

For protein extraction, the skin tissue was homogenized with T-PER^™^ tissue protein extraction reagent (cat. no. 78510; Pierce; Thermo Fisher Scientific, Inc.) supplemented with protease inhibitors (Roche Complete^™^, Mini Protease Inhibitor Cocktail; cat. no. 04693159001; Roche Applied Science). The protein concentration was measured with Quick Start Bradford 1X Dye Reagent (cat. no. 5000205; Bio-Rad Laboratories, Inc.) together with the Quick Start Bovine Serum Albumin Standard (cat. no. 5000207; Bio-Rad Laboratories, Inc.) in accordance with the manufacturer's protocol.

### CAT activity assay

CAT activity was measured colorimetrically as previously described (21). Briefly, 20 µl protein sample was incubated with 100 µl 65 mM H_2_O_2_ substrate at 37˚C for 1 min. The enzymatic reaction was terminated by adding 100 µl 32.4 mM ammonium molybdate, and the absorbance of the resulting yellow complex was subsequently measured at 405 nm using a microplate reader (VersaMax Microplate Reader).

### GSH detection

The concentration of GSH was determined colorimetrically according to a previously described protocol (15). Briefly, 20 µl protein sample or GSH standards (0.2-1.0 mM; cat. no. 70-18-8; MilliporeSigma) were mixed with 250 µl 0.1 M phosphate buffer (pH 7.6) and 50 µl 1 mM 5,5'-dithiobis-(2-nitrobenzoic acid) (cat. no. 69-78-3; MilliporeSigma). Following a 5-min incubation at room temperature, the absorbance was recorded at 412 nm using a microplate reader (VersaMax Microplate Reader).

### SOD activity assay

SOD activity was determined using a water-soluble tetrazolium salt (WST) assay kit (cat. no. S311; Dojindo Laboratories, Inc.) according to the manufacturer's instructions. Briefly, 20 µl protein sample was mixed with 200 µl WST working solution in a 96-well microplate. The reaction was initiated by adding 20 µl enzyme working solution to each well. After incubation at 37˚C for 20 min, the absorbance was measured at 450 nm using a microplate reader (VersaMax Microplate Reader).

### Lipid peroxidation (MDA assay)

Lipid peroxidation was quantified by measuring MDA levels via the thiobarbituric acid reactive substances assay, as previously described (21). Briefly, 150 µl protein sample or serially diluted 1,1,3,3-tetraethoxypropane (TEP) standards) was mixed with 125 µl each of 10% trichloroacetic acid, 5 mM EDTA and 8% SDS, along with 10 µl 500 ppm butylated hydroxytoluene. The mixture was incubated at room temperature for 10 min before adding 535 µl 0.6% TBA. The reaction mixture was then heated in a boiling water bath for 30 min. After cooling, the mixture was centrifuged at 10,381 x g at 25˚C for 5 min, and the absorbance of the supernatant was measured at 532 nm using a microplate reader (VersaMax Microplate Reader). MDA levels were calculated against a TEP standard curve.

### Histopathological examination

The dorsal skin was preserved in 10% neutral-buffered formalin at room temperature for 48 h, after which, the fixed tissues were washed with tap water for 30 min and processed for embedding in paraffin wax. The paraffin-embedded samples were subsequently cut into 4-µm sections and mounted onto silane-coated glass slides. The sections were deparaffinized, rehydrated and then stained with Mayer's hematoxylin (cat. no. 05-06002/L; Bio-Optica Milano Spa) and Eosin Y plus alcoholic solution (cat no. 05-11007/L; Bio-Optica Milano Spa). Finally, the sections were dehydrated, cleared, mounted and covered with a glass coverslip, and histopathology was observed under a light microscope. Epidermal and dermal thicknesses were accurately measured using ImageJ software version 1.32j (National Institutes of Health). For mast cell detection, after rehydration, the sections were stained with Giemsa solution (cat. no. RA-002-12; Biotechnical Co., Ltd.) as previously described (3), and the cells were observed and images were captured under a light microscope (Nikon Upright Microscope Eclipse Ni-U; Nikon Corporation). The mast cells were counted at four areas per 10x field.

### Immunohistochemical (IHC) staining

IHC staining was performed using the aforementioned formalin-fixed, paraffin-embedded dorsal skin tissue sections. Following deparaffinization and rehydration of the tissue sections, endogenous peroxidase activity was blocked with 3% H_2_O_2_ at room temperature for 10 min, and antigen retrieval was performed by incubating the sections in boiled Tris-EDTA buffer (pH 9.0) in a hot air oven at 99.5˚C for 20 min, followed by cooling at room temperature for 20 min. Non-specific binding was then blocked with a protein blocking solution (cat. no. ab64226; Abcam) for 1 h at room temperature. The sections were subsequently incubated for 3 h at room temperature with the following primary antibodies: Rat monoclonal anti-CD3 antibody (1:50 dilution; clone no. CD3-12; cat. no. ab11089; Abcam), rabbit monoclonal anti-CD4 antibody (1:50 dilution; cat. no. ab183685; Abcam) and rabbit monoclonal anti-proliferating cell nuclear antigen (PCNA) antibody (1:100 dilution; clone no. 3B22; cat.no. ZRB1442; MilliporeSigma). After washing with PBS containing 0.05% Tween-20 (pH 7.4), the sections were incubated for 1 h at room temperature with the corresponding secondary antibodies: Horseradish peroxidase (HRP)-conjugated goat anti-rat IgG (1:500 dilution; cat. no. AP136P; MilliporeSigma) and peroxidase AffiniPure^®^ goat anti-rabbit IgG (1:500 dilution; cat. no. 111-035-003; Jackson ImmunoResearch). Positive signals were visualized using NOVA Red (cat. no. SK-4800; Vector Laboratories, Inc.; Maravai LifeSciences) for 3 min at room temperature, followed by counterstaining with Mayer's hematoxylin for 20 sec.

For the detection of total and phosphorylated (p)-proteins, formalin-fixed, paraffin-embedded dorsal skin tissue sections prepared as aforementioned were incubated overnight at 4˚C with rabbit monoclonal anti-JAK2 antibody (cat. no. 3230; clone no. D2E12; 1:800 dilution; Cell Signaling Technology, Inc.) rabbit monoclonal anti-p-JAK2 (Y1007/Y1008) antibody (1:50 dilution; cat. no. ab32101; Abcam), rabbit polyclonal anti-JAK3 antibody (1:100 dilution; cat. no. E-AB-31846; Elabscience Bionovation Inc.), rabbit polyclonal anti p-JAK3 (Tyr785) antibody (1:100 dilution; cat. no. E-AB-21215; Elabscience Bionovation Inc.), rabbit polyclonal anti-STAT3 antibody (1:50 dilution; cat. no. ab31370; Abcam) and rabbit monoclonal anti-p-STAT3 (Tyr705) antibody (1:100 dilution; cat. no. 9145; Cell Signaling Technology, Inc.). Immunoreactivity for p-JAK2 (Y1007/Y1008) and p-STAT3 (Tyr705) was visualized using a rabbit-specific HRP/AEC IHC detection kit (cat. no. ab236468; Abcam), which includes the secondary antibody and HRP detection reagents, in accordance with the manufacturer's protocol. For the remaining antibodies, detection was performed using peroxidase-conjugated AffiniPure goat polyclonal anti-rabbit IgG secondary antibody (cat. no. 111-035-003; Jackson ImmunoResearch), diluted 1:500 and incubated in a humidified chamber at room temperature for 2 h. Positive signals were visualized using NOVA Red (cat. no. SK-4800; Vector Laboratories, Inc.; Maravai LifeSciences), in which positively stained cells appeared as red-brown coloration under light microscopy. For semi-quantitative analysis, images were acquired at 10x magnification using a light microscope (Nikon Upright Microscope Eclipse Ni-U; Nikon Corporation), and CD3^+^-, CD4^+^- and PCNA-positive cells were counted in four randomly selected areas per field.

### Lymphatic organ weight

The right axillary lymph nodes and spleens of the mice were removed, cleaned and weighed to calculate the lymphatic organ index using the following formula: Lymphatic organ index=organ weight (g)/mouse weight (g).

### Reverse transcription-quantitative PCR (RT-qPCR)

Total RNA was isolated from the skin, spleen and axillary lymph nodes using TRI Reagent^®^ (Cat no. TR118, Molecular Research Center, Inc.) in accordance with the manufacturer's protocol. Total RNA (1 µg) was converted to cDNA using iScript^™^ Reverse Transcription Supermix for RT-qPCR (cat. no. 1708840; Bio-Rad Laboratories, Inc.) according to the manufacturer's protocol under the following thermal cycling conditions: Priming at 25˚C for 5 min, RT at 46˚C for 20 min and RT inactivation at 95˚C for 1 min. All RT-qPCR assays were performed on the QIAquant Real-Time PCR Thermal Cycler (Qiagen, Inc.) with a SensiFAST^™^ SYBR^®^ No-ROX kit (cat. no. BIO-98050; Bioline; Meridian Bioscience). The thermocycling conditions were as follows: Initial polymerase activation at 95˚C for 2 min, followed by 40 cycles of denaturation at 95˚C for 5 sec and annealing/extension at 65˚C for 20 sec. The relative expression levels of the target genes, including *IL-1b*, *IL-6, keratin* 6 (*K6*), *K16*, *K17*, *S100A8*, *S100A9*, *JAK1*, *JAK2*, *JAK3*, *STAT1*, *STAT2*, *STAT3*, *Nrf2*, *FLG*, *LOR*, *Cu/Zn-SOD*, *Mn-SOD*, *CAT* and *GSH-Px*, were normalized to the levels of *β-actin* and were calculated with the 2^-ΔΔCq^ method (22). The primer sequences of the target genes are shown in Table I.

### Western blot analysis

Total protein from the dorsal skin was extracted by homogenizing with tissue protein extraction reagent (T-PER tissue protein extraction reagent) supplemented with a protease inhibitor (Roche Complete, Mini Protease Inhibitor Cocktail). Homogenized proteins were then centrifuged at 12,000 x g for 10 min at room temperature and the supernatant was collected. The protein concentration was quantified on a NanoDrop 2000 spectrophotometer (NanoDrop; Thermo Fisher Scientific, Inc.). Protein samples (40 µg/each) were then loaded and separated by sodium dodecyl sulfate-polyacrylamide gel electrophoresis on 12.5% gels and transferred onto a nitrocellulose (NC) membrane (Amersham^™^ Protran^™^ Premium 0.45 µm NC; Cytiva). The membrane was washed twice (5 min each) with TBST (100 mM Tris-base, 150 mM NaCl, 0.1% Tween 20) and then incubated with 2% bovine serum albumin (cat. no. BSA-1S; Capricorn Scientific GmbH) in TBST for 2 h at room temperature. After being washed, the membrane was incubated with rabbit monoclonal antibody anti-Nrf2 (1:1,000 dilution; cat. no. 12721; Cell Signaling Technology, Inc.) overnight at 4˚C. To remove excessive antibody, the membrane was washed and then incubated with a peroxidase AffiniPure^®^ goat anti-rabbit IgG secondary antibody (1:5,000 dilution; cat. no. 111-035-003; Jackson ImmunoResearch) for 2 h at room temperature. Finally, the protein was detected using SuperKine^™^ West Pico PLUS chemiluminescent substrate (cat. no. BMU101-EN; Abbkine Scientific Co., Ltd.) and visualized with a chemiluminescence detection system. Images were captured with an ImageQuant^™^ 800 biomolecular imager (Amersham; Cytiva). The protein bands were semi-quantified using ImageJ software version 1.32j and graphically presented using GraphPad software (version 10.5.0.774; Dotmatics).

### Statistical analysis

Data are presented as the mean ± SD or median with IQR. GraphPad software (version 10.5.0.774) was used for statistical analysis and graph generation. Differences between the mean values of each experimental group were assessed using one-way analysis of variance with Tukey's multiple comparisons test. Differences between the median values were analyzed using the Kruskal-Wallis test followed by Dunn's multiple comparison post hoc test. P<0.05 was considered to indicate a statistically significant difference.

## Results

### PLO ameliorates psoriatic symptoms in a mouse model of IMQ-induced psoriasis

**Figure 1:**
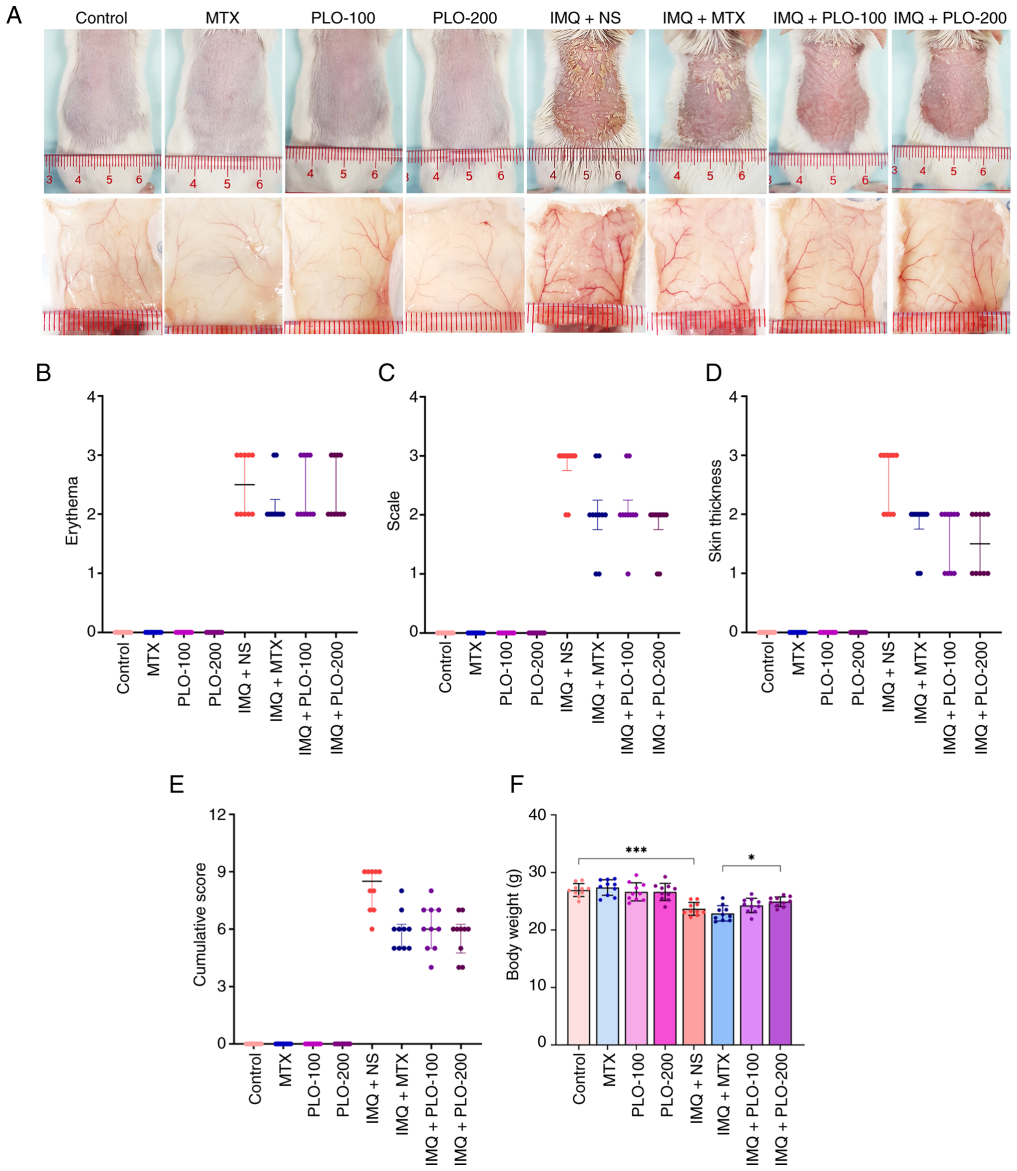
PLO reduces psoriatic lesions on the dorsal skin of mice. (A) Representative images of gross pathological morphology of dorsal skin (upper panel) and retracted skin showing subcutaneous vasculature (lower panel). PASI scores for (B) erythema, (C) scaling and (D) skin thickness, and (E) total PASI score were evaluated on day 8 prior to termination (n=10 mice/group). Data are presented as median (black horizontal line) with interquartile range and statistical significance was determined using the Kruskal-Wallis test followed by Dunn's multiple comparison post hoc test. No statistically significant differences were observed. (F) Body weight of mice in all groups was measured on day 8. Data are presented as the mean ± SD and statistical significance was determined by one-way ANOVA. ^*^P<0.05, ^***^P<0.001. IMQ, imiquimod; MTX, methotrexate; NS, normal saline; PASI, psoriasis area and severity index; PLO, pink lotus flower oil.

The effects of PLO treatment on IMQ-induced psoriasis were investigated. Compared with the control group and consistent with the MTX group, the PLO-100 and PLO-200 groups showed no notable differences in any detected parameters, including skin morphology, erythema, scaling, thickening and neovascularization in the subcutaneous tissue (Fig. 1; Fig. S1). After daily IMQ treatment for 7 days and termination on day 8, the mice in the IMQ + NS group developed psoriasis-like symptoms, including erythema, scaling and skin thickening, together with increased neovascularization, in the subcutaneous tissue of the dorsal skin (Fig. 1A). The severity of these symptoms was assessed, and the results are expressed as the PASI score in Fig. 1B-E. Related skin manifestations tended to be ameliorated in the IMQ + PLO-100 and IMQ + PLO-200 groups, as reflected by lower PASI scores, which were consistent with the effects observed in the MTX-treated group; however, these reductions did not reach statistical significance. Notably, body weight was significantly decreased following IMQ treatment compared with that in the control group. Furthermore, the PLO-treated groups (100 and 200 mg/kg body weight) showed a trend toward improved body weight compared with the IMQ + NS group, but these differences were not statistically significant. However, the IMQ + PLO-200 group showed a significant increase in body weight compared with in the IMQ + MTX group (Fig. 1F).

### PLO decreases histopathological changes, and keratinocyte proliferation and differentiation

**Figure 2:**
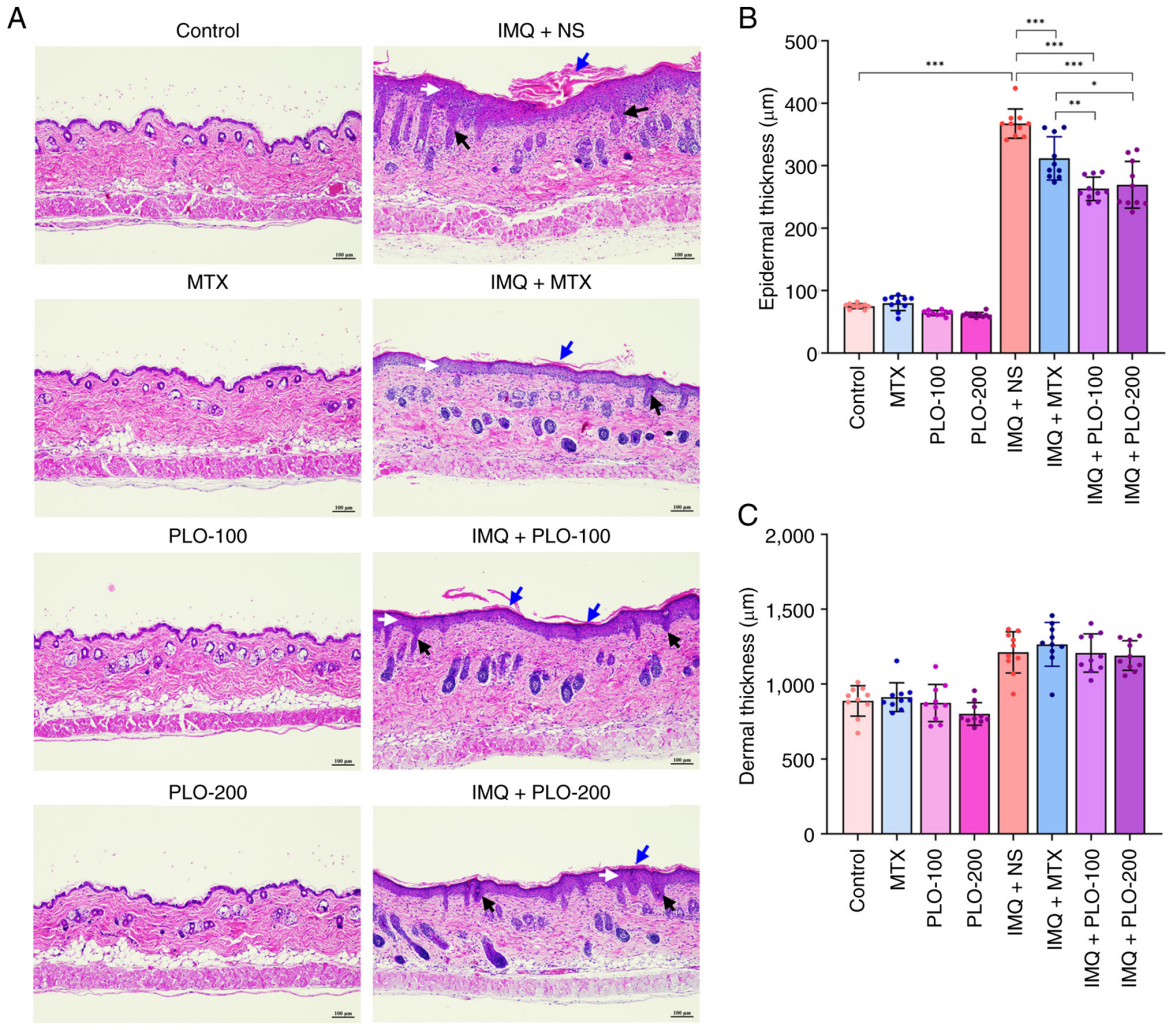
PLO ameliorates epidermal hyperplasia and regulates keratinocyte differentiation in mice with IMQ-induced psoriasis. (A) Representative hematoxylin and eosin-stained skin sections showing alleviation of epidermal hyperplasia (white arrow) parakeratosis (blue arrow) and elongated rete ridge (black arrow). Images were captured at x10 magnification (scale bar, 100 µm). (B) Semi-quantification of epidermal thickness in all groups (n=10 mice/group). (C) Semi-quantification of dermal thickness in all groups (n=10 mice/group). Data are presented as the mean ± SD and statistical significance was determined by one-way ANOVA. ^*^P<0.05, ^**^P<0.01, ^***^P<0.001. IMQ, imiquimod; MTX, methotrexate; NS, normal saline; PLO, pink lotus flower oil.

As shown in Fig. 2, histopathological analysis revealed marked epidermal hyperplasia (white arrow), parakeratosis (blue arrow) and elongated rete ridges (black arrow) consistent with typical psoriatic pathology in the IMQ + NS group compared with the normal skin histology observed in the control group. The IMQ + MTX group showed partial restoration of epidermal architecture, with a moderate reduction in epidermal thickness. Notably, the IMQ + PLO-100 and IMQ + PLO-200 groups exhibited significantly improved skin histology (Fig. 2A and B). Although no difference in dermal layer thickening was observed (Fig. 2C), the IMQ + PLO groups exhibited a thinner epidermis and better-restored epidermal stratification than the IMQ + NS group.

**Figure 3:**
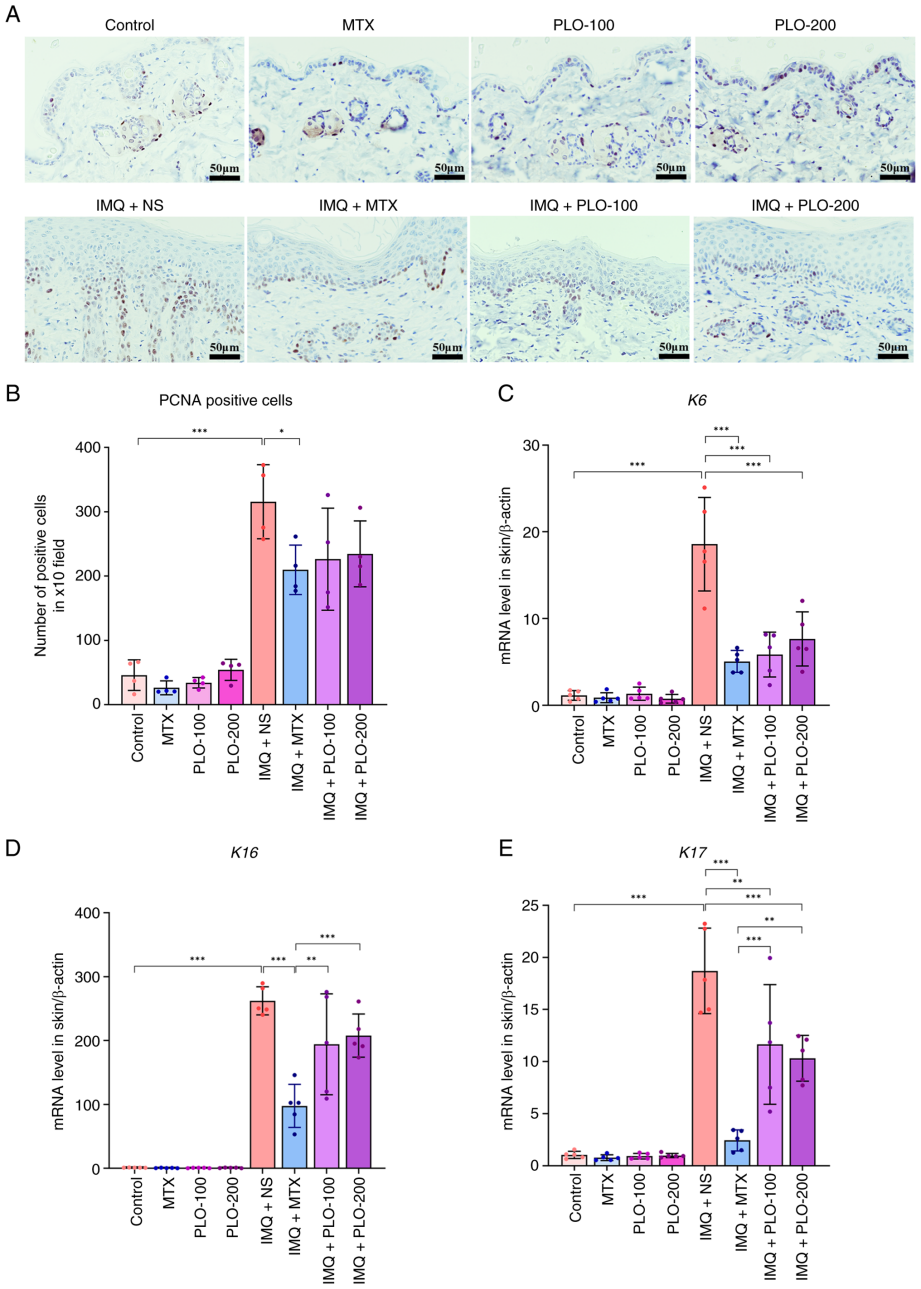
PLO reduces keratinocyte proliferation and downregulates psoriasis-associated gene expression in mice with IMQ-induced psoriasis. (A) Representative immunohistochemical staining for PCNA in dorsal skin sections. Images were captured at x10 magnification (scale bar, 100 µm). (B) Semi-quantification of PCNA-positive cells in epidermal regions; positive cells were counted in 5 random microscopic fields per section (n=4 mice/group). Relative mRNA expression levels of genes encoding (C) *K6*, (D) *K16* and (E) *K17* detected by reverse transcription-quantitative PCR (n=5 mice/group). Data are presented as the mean ± SD and statistical significance was determined by one-way ANOVA. ^*^P<0.05, ^**^P<0.01, ^***^P<0.001. IMQ, imiquimod; *K*, keratin; MTX, methotrexate; NS, normal saline; PCNA, proliferating cell nuclear antigen; PLO, pink lotus flower oil.

IHC staining confirmed that the IMQ + NS group exhibited epidermal hyperproliferation, as indicated by the increased numbers of PCNA-positive cells in the basal and suprabasal layers of the epidermis (Fig. 3A and B). Notably, the IMQ + PLO groups exhibited markedly decreased numbers of PCNA-positive cells, and the reduction in PCNA-positive cells was comparable to that observed in the IMQ + MTX group. In addition, increased expression of keratinocyte proliferation markers, such as PCNA (23), is associated with aberrant induction of *K6*, *K16* and *K17*, which regulate keratinocyte proliferation, migration and inflammation (24-26). These markers are commonly used to confirm successful establishment of IMQ-induced psoriasis-like mouse models (3,27). RT-qPCR analysis revealed that the expression levels of *K6* and *K17* were significantly increased in the IMQ + NS groups compared with those in the control, IMQ + MTX and IMQ + PLO treated groups (Fig. 3C and E). Furthermore, *K16* expression was significantly reduced in the IMQ + MTX group compared with in the IMQ + NS group and IMQ + PLO treated groups; however, although a decreasing trend was observed in the IMQ + PLO groups relative to the IMQ + NS group, the differences did not reach statistical significance (Fig. 3D). Collectively, these findings suggested that PLO mitigates IMQ-induced epidermal hyperplasia and keratinocyte hyperproliferation by downregulating key markers of abnormal differentiation, thereby restoring epidermal integrity.

### PLO attenuates proinflammatory cytokine expression and improves the immune microenvironment in mice with IMQ-induced psoriasis

**Figure 4:**
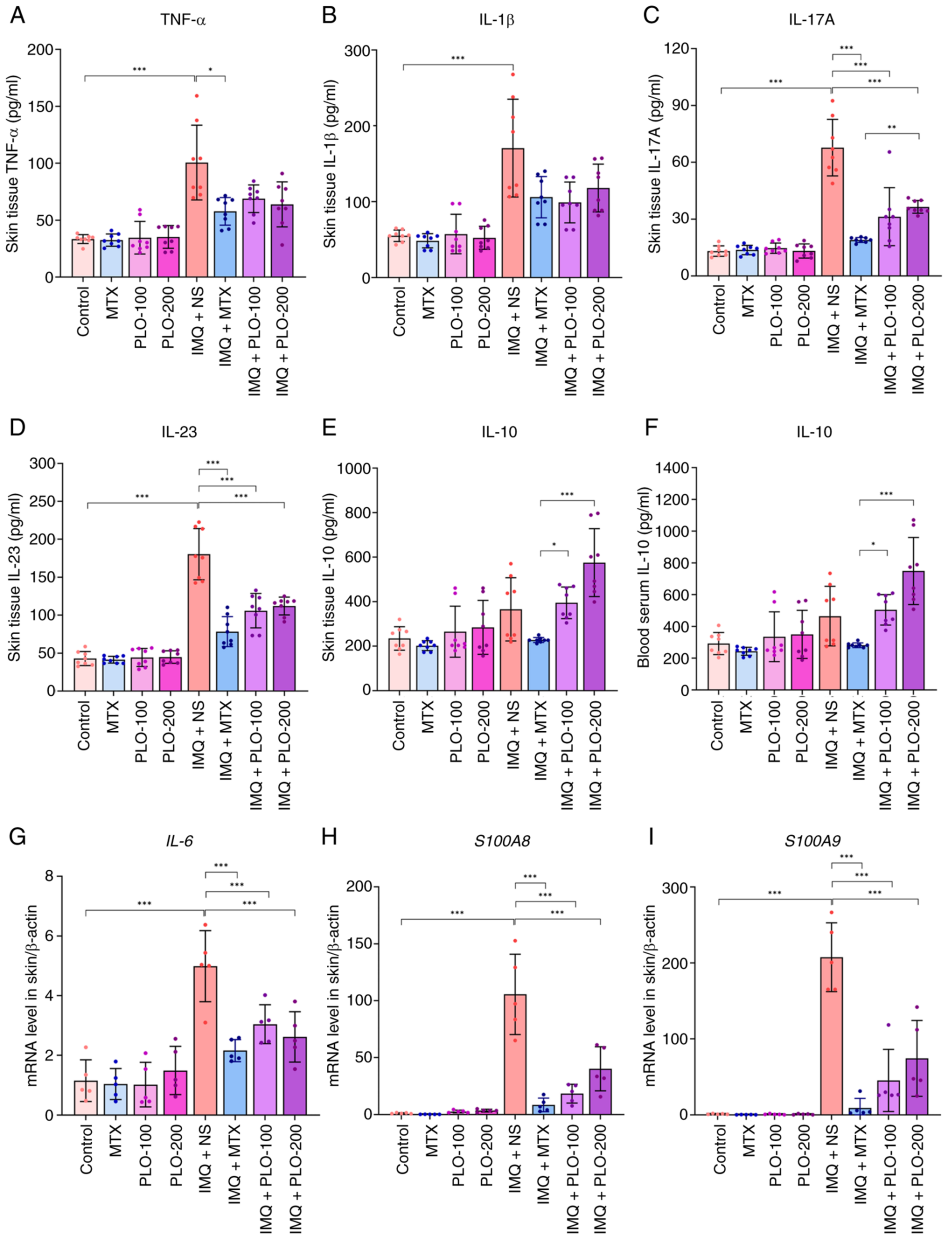
PLO reduces skin inflammation in mice with IMQ-induced psoriasis. Cytokine levels in skin tissue and serum were evaluated by ELISA (n=8 mice/group) and RT-qPCR (n=5 mice/group). ELISA results of (A) TNF-α, (B) IL-1β, (C) IL-17A and (D) IL-23 levels in the skin tissue, and IL-10 levels in the (E) skin tissue and (F) serum. RT-qPCR results of the relative mRNA expression levels of (G) *IL-6*, (H) *S100A8* and (I) *S100A9*. Data are presented as the mean ± SD and statistical significance was determined by one-way ANOVA. ^*^P<0.05, ^**^P<0.01, ^***^P<0.001. IMQ, imiquimod; MTX, methotrexate; NS, normal saline; PLO, pink lotus flower oil; RT-qPCR, reverse transcription-quantitative PCR.

The levels of key proinflammatory and anti-inflammatory cytokines, as well as inflammatory markers, were assessed in skin tissues and serum from mice with IMQ-induced psoriasis treated with PLO, in order to elucidate its anti-inflammatory efficacy. ELISA results showed a decreasing trend in TNF-α and IL-1β levels in the dorsal skin of the IMQ + PLO-100 and IMQ + PLO-200 groups compared with in the IMQ + NS group; however, these differences were not statistically significant (Fig. 4A and B). Notably, the levels of IL-17A and IL-23 in the dorsal skin were significantly lower in the IMQ + PLO-100 and IMQ + PLO-200 groups than those in the IMQ + NS group (Fig. 4C and D). By contrast, the levels of IL-10 in both dorsal skin and serum showed an increasing trend in the IMQ + PLO-200 group compared with in the IMQ + NS group; however, this difference was not statistically significant (Fig. 4E and F). Gene analysis revealed that the mRNA levels of *IL-6, S100A8* and *S100A9* were significantly decreased in the IMQ + PLO-100 and IMQ + PLO-200 groups compared with in the IMQ + NS group, with effects comparable to those observed in the IMQ + MTX group (Fig. 4G-I).

**Figure 5:**
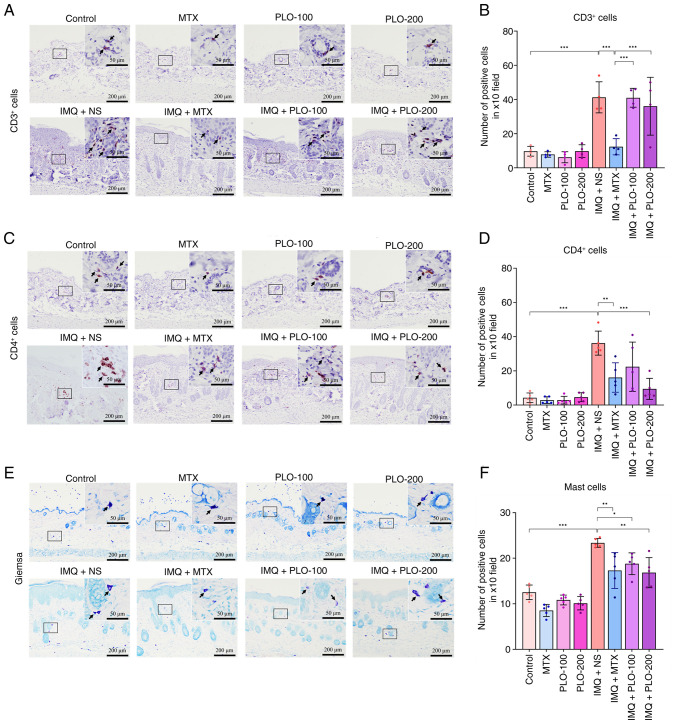
Distribution of inflammatory cells, including CD3^+^, CD4^+^ and mast cells, in dorsal skin sections of mice with IMQ-induced psoriasis. (A) Representative immunohistochemical staining for CD3^+^, indicated by black arrow, and (B) semi-quantification of CD3^+^ cells in epidermal regions (n=4 mice/group). (C) Representative immunohistochemical staining for CD4^+^, indicated by black arrow, and (D) semi-quantification of CD4^+^ cells in epidermal regions (n=5 mice/group). (E) Representative Giemsa staining of mast cells, indicated by black arrow, and (F) semi-quantification of mast cells in epidermal regions (n=5 mice/group). Images were captured at x10 magnification (scale bar, 100 µm), and integrated optical densities were measured in five random fields per section. Data are presented as the mean ± SD and statistical significance was determined by one-way ANOVA. ^*^P<0.05, ^**^P<0.01, ^***^P<0.001. IMQ, imiquimod; MTX, methotrexate; NS, normal saline; PLO, pink lotus flower oil.

Increased immunoreactive staining indicative of CD3^+^ cell infiltration was significantly observed in the IMQ + NS group compared with that in the control group. Additionally, a significantly decrease and a decreasing trend were observed in the IMQ + MTX and IMQ + PLO-200 groups compared with in the IMQ + NS group, respectively (Fig. 5A and B). Although CD3^+^ cell infiltration showed no significant differences between the IMQ + NS and IMQ + PLO groups, CD4^+^ cell infiltration was significantly increased in the IMQ + NS group compared with in the control group, and by contrast, CD4^+^ cell infiltration was significantly reduced in the IMQ + PLO-200 group compared with in the IMQ + NS group (Fig. 5C and D). Additionally, Giemsa staining showed that mast cell infiltration was significantly increased in the IMQ + NS group compared with in the control group, whereas significant reductions were observed in the IMQ + PLO-100 and IMQ + PLO-200 groups compared with in the IMQ + NS group (Fig. 5E and F). Collectively, these results demonstrated that PLO could mitigate IMQ-induced psoriatic inflammation by targeting key inflammatory cytokines and inflammatory cell infiltration.

### PLO suppresses activation of JAK2 and the JAK3/STAT3 pathway in IMQ-induced psoriasis

**Figure 6:**
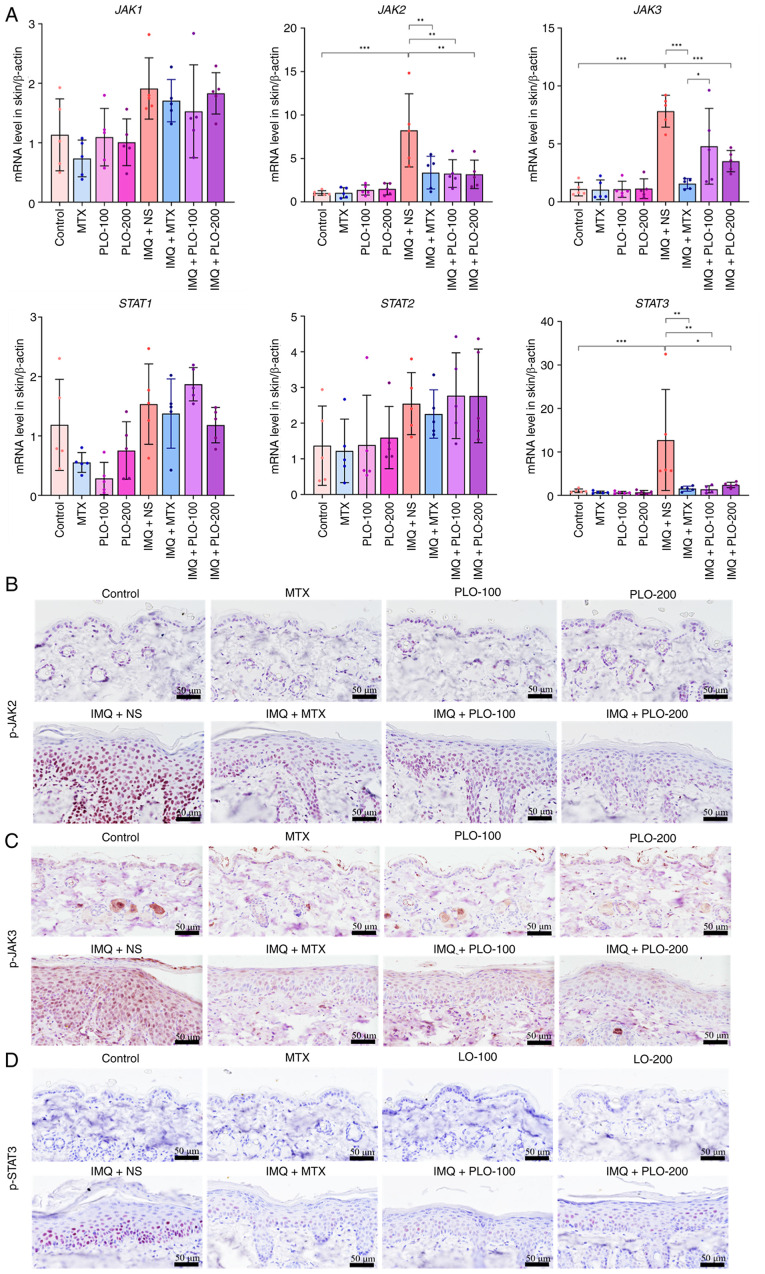
PLO reduces *JAK/STAT* expression in mice with IMQ-induced psoriasis. (A) Reverse transcription-quantitative PCR analysis of mRNA expression levels of *JAK1*, *JAK2*, *JAK3*, *STAT1*, *STAT2* and *STAT3* in the dorsal skin. Representative immunohistochemical staining showing the expression of (B) p-JAK2, (C) p-JAK3 and (D) p-STAT3. Images were captured at x40 magnification (scale bar, 50 µm). Data are presented as the mean ± SD (n=5 mice/group) and statistical significance was determined by one-way ANOVA. ^*^P<0.05, ^**^P<0.01, ^***^P<0.001. IMQ, imiquimod; MTX, methotrexate; NS, normal saline; p-, phosphorylated; PLO, pink lotus flower oil.

As shown in Fig. 6A, RT-qPCR analysis revealed that the mRNA levels of *JAK2* and *STAT3* were significantly increased in the IMQ + NS group compared with those in the control, IMQ + MTX, IMQ + PLO-100 and IMQ + PLO-200 groups. Additionally, the mRNA expression levels of *JAK3* were significantly increased in the IMQ + NS groups compared with in the control group, whereas *JAK3* mRNA expression showed a decreasing trend in the IMQ + PLO-100 group, and was significantly decreased in the IMQ + PLO-200 group compared with in the IMQ + NS group. IHC staining of p-JAK2, p-JAK3 and p-STAT3 showed that the numbers of p-JAK2-, p-JAK3- and p-STAT3-expressing cells were notably lower in the epidermis of the IMQ + PLO-100 and IMQ + PLO-200 groups than those in the IMQ + NS group (Fig. 6B-D). IHC analysis further demonstrated a decreasing trend in the numbers of total JAK2-, JAK3-, and STAT3-positive cells in the IMQ + MTX, IMQ + PLO-100, and IMQ + PLO-200 groups compared with the IMQ + NS group, although the reduction was less evident than that observed in the phosphorylated proteins (Fig. S2).

### PLO promotes cutaneous antioxidants and improves the skin barrier in IMQ-induced psoriasis

**Figure 7:**
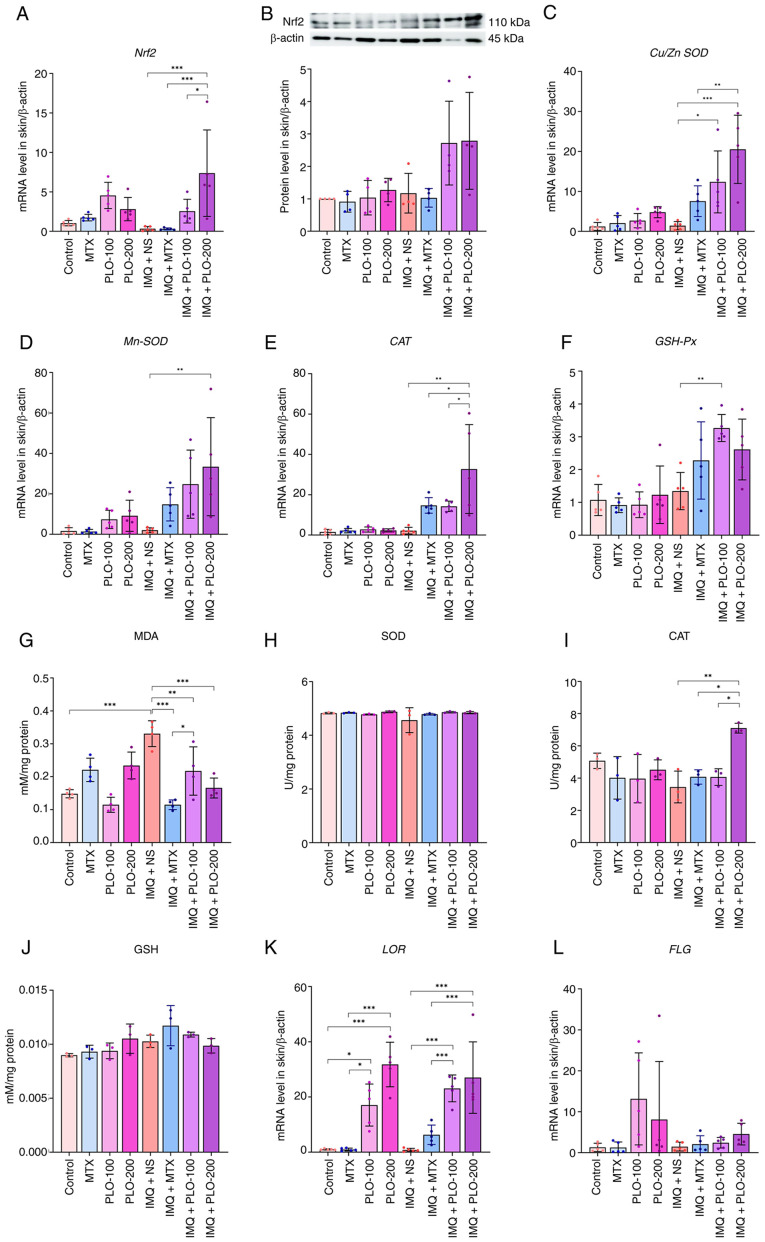
Effects of PLO on oxidative damage in mice with IMQ-induced psoriasis. (A) Reverse transcription-quantitative PCR analysis of *Nrf2* mRNA expression (n=5 mice/group). (B) Relative protein expression of Nrf2 in skin tissues examined by western blotting (n=4 mice/group). Relative mRNA expression levels of antioxidant enzymes (C) *Cu/Zn*-*SOD*, (D) *Mn-SOD*, (E) *CAT* and (F) *GSH-Px* (n=5 mice/group). Protein levels of (G) MDA, (H) SOD, (I) CAT and (J) GSH (n=4 mice/group). mRNA expression levels of skin barrier gene (K) *LOR* and (L) *FLG* (n=5 mice/group). Data are presented as the mean ± SD and statistical significance was determined by one-way ANOVA. ^*^P<0.05, ^**^P<0.01, ^***^P<0.001. CAT, catalase; *FLG*, filaggrin; GSH, reduced glutathione; *GSH-Px*, glutathione peroxidase; IMQ, imiquimod; *LOR*, loricrin; MDA, malondialdehyde; MTX, methotrexate; *Nrf2*, nuclear factor erythroid 2-related factor 2; NS, normal saline; PLO, pink lotus flower oil; SOD, superoxide dismutase.

The mRNA and protein expression levels of key factors regulating cellular defenses against stress were assessed in the dorsal skin of IMQ-treated mice to evaluate the impact of PLO on oxidative stress modulation in psoriasis-like inflammation. The mRNA expression levels of *Nrf2* were increased in the IMQ + PLO-100 group and significantly increased in the IMQ + PLO-200 group compared with those in the IMQ + NS group (Fig. 7A). Notably, the mRNA expression levels of *Nrf2* also increased in the PLO-100 and PLO-200 groups compared with in the IMQ + NS group. The protein expression levels of Nrf2 were also increased, but the difference between the IMQ + PLO and IMQ + NS groups was not significant (Fig. 7B). Additionally, the mRNA expression levels of *Cu/Zn-SOD*, *Mn-SOD* and *CAT* were significantly increased in the IMQ + PLO-200 group compared with in the IMQ + NS group, reflecting enhanced dismutation of superoxide radicals (Fig. 7C-E). Notably, *CAT* expression was markedly elevated in the IMQ + PLO-200 group compared with in the IMQ + NS, IMQ + MTX, and IMQ + PLO-100 groups (Fig. 7E). In parallel, the expression of *GSH-Px*, a critical enzyme involved in the reduction of lipid hydroperoxides (17), was significantly upregulated in the IMQ + PLO-100 group compared with in IMQ + NS group, and showed an increasing trend in the IMQ + PLO-200 group compared with in the IMQ + NS and IMQ + MTX groups (Fig. 7F). Notably, MDA levels were significantly elevated in the IMQ + NS group compared with those in the control, IMQ + MTX, IMQ + PLO-100 and IMQ + PLO-200 groups (Fig. 7G). This finding corresponded with the decreased protein expression of SOD and CAT in the IMQ + NS group (Fig. 7H and I), whereas CAT protein levels were significantly increased in the IMQ + PLO-200 group compared with in the IMQ + NS group (Fig. 7I). However, the protein levels of SOD and GSH did not differ significantly among the groups (Fig. 7I and J).

Inhibiting oxidative stress through the activation of Nrf2 should upregulate the expression of epidermal barrier proteins, including LOR and FLG (28). RT-qPCR was performed to measure the mRNA expression levels of *LOR* and *FLG*. The mRNA expression levels of *LOR* were significantly lower in the IMQ + NS group than in the IMQ + PLO-100 and IMQ + PLO-200 groups (Fig. 7K). Furthermore, the mRNA expression levels of *FLG* were higher in the IMQ + PLO-100 and IMQ + PLO-200 groups than in the IMQ + NS group, but the difference was not statistically significant (Fig. 7L). These results indicated that the antioxidative effect of PLO contributes to its broader anti-inflammatory action and therapeutic potential in oxidative stress-mediated skin disorders.

### PLO ameliorates IMQ-induced systemic inflammation and does not cause toxicity to other vital organs

**Figure 8:**
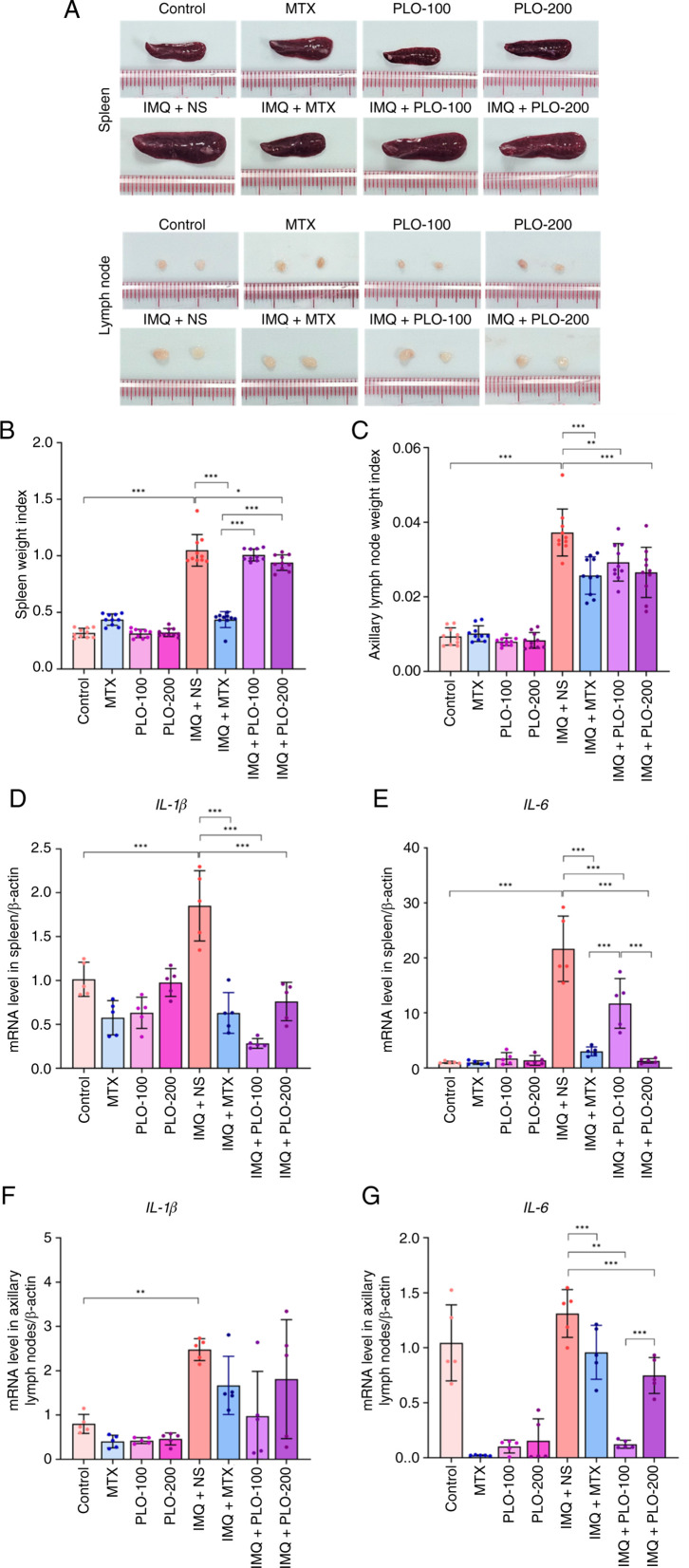
PLO reduces systemic inflammation. (A) Representative images of the spleen and lymph nodes on day 8 after treatment. (B) Relative spleen weight in the control and experimental groups (n=10). (C) Relative lymph node weight in the control and experimental groups (n=10). mRNA expression levels of (D) *IL-1β* and (E) *IL-6* in the spleen (n=5). mRNA expression levels of (F) *IL-1β* and (G) *IL-6* in the right axillary lymph nodes (n=5). Data are presented as the mean ± SD and statistical significance was determined by one-way ANOVA. ^*^P<0.05, ^**^P<0.01, ^***^P<0.001. IMQ, imiquimod; MTX, methotrexate; NS, normal saline; PLO, pink lotus flower oil.

Psoriasis is associated with systemic immune activation, often evidenced by hypertrophy of secondary lymphoid organs such as the spleen and lymph nodes (3,29). Spleens and axillary lymph nodes in the IMQ-induced model were collected and evaluated for weight indices and inflammatory cytokine expression. As shown in Fig. 8A-C, the IMQ + NS group exhibited significantly larger spleen and lymph node weights than the control group. However, the IMQ + PLO groups, particularly the IMQ + PLO-200 group, exhibited smaller spleen sizes and lower spleen indices than the IMQ + NS group (Fig. 8A and B). RT-qPCR analysis revealed that the expression levels of *IL-1b* and *IL-6* in splenic tissue were significantly lower in the IMQ + PLO-100 and IMQ + PLO-200 groups than those in the IMQ + NS group (Fig. 8D and E). Furthermore, the axillary lymph nodes decreased in size and exhibited significantly reduced relative weight in the IMQ + PLO groups than that in the IMQ + NS group (Fig. 8A and C). The mRNA expression levels of *IL-1b* were lower in the lymph node samples of the IMQ + PLO groups compared with in the IMQ + NS group, although the difference was not significant (Fig. 8F). In addition, *IL-6* mRNA expression was significantly lower in the lymph node samples of the IMQ + PLO groups than that in the IMQ + NS group (Fig. 8G). Histopathological examination of major vital organs revealed no apparent inflammation or injury in either PLO-treated normal mice or IMQ-treated mice (Figs. S3 and S4), indicating that PLO treatment did not cause adverse effects. Taken together, these findings suggest that PLO administration alleviates systemic inflammation and exhibits a favorable safety profile for psoriasis treatment.

## Discussion

PLO treatment exerts neuroprotective effects by increasing antioxidant activity (15) and alleviates free fatty acid-induced steatosis in HepG2 cells by inhibiting inflammatory responses (19). The present study investigated the potential therapeutic effect of PLO on a mouse model of IMQ-induced psoriasis. IMQ treatment in mice induced characteristic symptoms of psoriasis, including erythema, scaling, skin thickening and an elevated PASI score. Oral administration of PLO reduced erythema, skin thickening and scaling in the skin of the IMQ-treated mice.

Natural products exert anti-psoriatic effects through multi-targeted mechanisms, including anti-inflammatory and immunomodulatory activities (30), consistent with the central role of immune dysregulation in psoriasis. The anti-psoriasis-like effects of PLO may be attributed to its lipid-derived constituents identified by GC-MS, particularly unsaturated fatty acid derivatives such as linoleic acid ethyl ester (16). These esterified compounds may undergo *in vivo* hydrolysis to release free fatty acids that regulate skin homeostasis and inflammation (31,32). Linoleic acid and its derivatives suppress key inflammatory pathways, including NF-κB and JAK/STAT signaling, thereby reducing pro-inflammatory cytokines such as IL-1β, IL-6 and IL-17. This is relevant to IMQ-induced psoriasis-like inflammation driven by the IL-23/IL-17 axis, which promotes keratinocyte hyperproliferation and immune cell infiltration (33-36). Linoleic acid also supports epidermal barrier integrity via ceramide synthesis (31). By contrast, saturated fatty acid derivatives such as palmitic acid ethyl ester are less likely to mediate these effects, as palmitic acid can promote NF-κB-driven inflammation, although context dependent (37-39). Overall, the therapeutic effects of PLO likely arise from synergistic interactions among its lipid constituents, particularly unsaturated fatty acids, resulting in attenuated inflammatory responses.

One of the key histological hallmarks of psoriasis is the excessive proliferation of keratinocytes in response to inflammation with aberrant terminal differentiation, features reflected by upregulation of proliferation-associated proteins, leading to epidermal thickening (3,26,40). The epidermal basal and suprabasal layers show markedly increased proliferation of keratinocyte markers, PCNA and Ki67(23), which aligns with the aberrant induction of *K6, K16* and *K17*, which serve critical roles in modulating keratinocyte proliferation, migration and inflammation (24-26). These markers have been used as key biomarkers in IMQ-induced mouse psoriasis and experimental treatments (3,27). In the present study, PLO treatment significantly reduced keratinocyte proliferation and decreased keratin expression, suggesting a reduced severity of skin inflammation. Although epidermal hyperplasia was significantly reduced, dermal thickness did not differ significantly among groups. This may reflect distinct pathological dynamics, as epidermal changes occur rapidly, whereas dermal remodeling, such as extracellular matrix changes, fibroblast activation and vascular alterations, progresses more slowly (1,8).

Dysregulation of the IL-23/IL-17 signaling pathway drives the development and progression of psoriasis (1,5,41,42). IL-1β and IL-23 are crucial for the development and proliferation of Th17 cells (43), which produce cytokines such as IL-17 and TNF-α that promote dermal immune cell infiltration and activation (44-46), as well as proinflammatory S100 molecules (S100A8 and S100A9) that further amplify skin inflammation (47). Accordingly, the current study measured key cytokines involved in psoriasis pathogenesis, including *IL-6*, IL-1β, IL-17A, IL-23, *S100A8* and *S100A9*. PLO treatment markedly reduced the levels of these proinflammatory mediators, indicating attenuation of IMQ-induced skin inflammation. In addition, the infiltration of immune cells, including CD4^+^ T cells and mast cells, were decreased in the PLO-treated mice. Consistent with previous studies, reductions in CD4^+^ T and mast cells were associated with decreased disease severity (3,27). Notably, although the infiltration of CD4^+^ T and mast cells was significantly reduced, total CD3^+^ T cell numbers did not differ significantly between the IMQ + NS and IMQ + PLO groups. This result may reflect the heterogeneity of T-cell populations in psoriasis. CD3^+^ is a pan-T-cell marker encompassing CD4^+^, CD8^+^ and γδ T cells. In IMQ-induced models, IL-17-producing γδ T cells are key drivers of early inflammation and may persist despite reductions in other subsets (6,41,44). Thus, selective reduction of CD4^+^ T cells may occur without a substantial decrease in total CD3^+^ cells. Additionally, remaining CD3^+^ cells may include resident or regulatory T cells present during the resolution phase (42). Targeting inflammatory cytokines or Th17 responses reduces CD4^+^ infiltration with limited effects on total CD3^+^ cells (3,27). Collectively, these findings suggest that PLO selectively modulates specific inflammatory T-cell subsets rather than broadly depleting all T cells in psoriatic lesions.

IL-17 and IL-23 mediate immune activation and inflammatory responses via the JAK/STAT signaling pathway (7,35,44,48,49). Inhibition of the JAK/STAT pathway alleviates psoriatic inflammation and suppression of JAK2/STAT3 signaling reduces IMQ-induced pathology in various mouse model studies (3,27,36,50). In the present study, the mRNA expression levels of *JAK2*, *JAK3* and *STAT3* were reduced in the PLO-treated mice with IMQ-induced psoriasis, corresponding to the number of keratinocytes expressing p-JAK2 and p-STAT3. These findings suggest that PLO exerts its anti-inflammatory effects, at least in part, by inhibiting the JAK/STAT signaling cascade, a key driver of psoriatic inflammation.

Nrf2 is a key regulator of the cellular antioxidant defense system. Under oxidative stress, Keap1 loses its ability to inhibit Nrf2, allowing Nrf2 to move into the nucleus and activate genes with antioxidant response elements in their promoters (40). Mice lacking Nrf2 show worsened psoriasis-like symptoms, whereas suppression of oxidative stress and inflammation through activation of the Keap1/Nrf2 pathway alleviates IMQ-induced psoriasis in mice (40,51,52). Additionally, oxidative stress decreases the expression of key skin barrier-related proteins, including LOR and FLG (13). Decreased expression of these proteins has been associated with lesional and non-lesional skin of patients with psoriasis (53). Therefore, the inhibition of oxidative stress and increase in antioxidants can alleviate inflammation-related diseases by suppressing the overproduction of cytokine-driven inflammatory responses (54,55). In the present study, the IMQ + PLO-treated mice had upregulated mRNA expression levels of *Nrf2* and antioxidant genes, including *Cu/Zn-SOD*, *Mn-SOD*, *CAT* and *GSH-Px*, decreased MDA levels and increased CAT levels. These results correspond to a previous report, which demonstrated that treating IMQ mice with rutin promotes the expression of Nrf2 and antioxidant proteins, alleviating IMQ-induced psoriasis in mice (56). Although PLO enhanced antioxidant gene expression and reduced lipid peroxidation, no significant changes were observed in SOD and GSH protein levels. This discrepancy likely reflects the complex regulation of antioxidant systems, where transcriptional upregulation does not necessarily translate into proportional protein increases due to post-transcriptional and post-translational controls (11,54). Additionally, antioxidant enzymes are tightly maintained within a narrow physiological range to preserve redox homeostasis, rendering oxidative damage markers, such as MDA, more sensitive indicators of oxidative stress. Activation of Nrf2 may also preferentially enhance antioxidant capacity without markedly altering enzyme abundance (10,12,51), whereas biological variability and limited sample size may further obscure subtle protein-level differences. Collectively, these findings suggest that PLO mitigates oxidative stress primarily by enhancing antioxidant defense capacity and reducing oxidative damage, rather than by substantially increasing antioxidant enzyme levels.

Furthermore, PLO restored the expression of the key skin barrier gene *LOR*, whereas *FLG* expression was not significantly affected. Together, these results suggest that PLO activates Nrf2, enhances antioxidant defenses, reduces inflammation and promotes skin barrier restoration. However, a limitation of the current study is that the specific PLO components responsible for its anti-psoriatic activity (for example, palmitic acid ethyl ester and linoleic acid ethyl ester) remain to be fully confirmed and elucidated.

In conclusion, the present study reported that PLO can alleviate IMQ-induced psoriasis in mice by decreasing inflammatory cell infiltration and cytokine production through inhibition of the IL-23/IL-17 axis via modulation of the JAK2/JAK3/STAT3 pathway, and by inhibiting oxidative stress through activation of downstream signaling of the antioxidant transcription factor Nrf2. Therefore, PLO may be considered an effective therapeutic agent for psoriasis management. Nonetheless, further comprehensive studies are warranted to evaluate the long-term toxicity and safety of PLO and support its future clinical applications.

## Supplementary Material

PLO improves IMQ-induced psoriatic symptoms in mice. (A) Representative clinical images of dorsal skin on days 1, 3, 5, 7 and 8 following IMQ treatment. PASI scores, including (B) erythema, (C) scaling and (D) skin thickness, and (E) cumulative PASI score were evaluated daily. (F) Body weight of mice in all groups was measured daily (n=10). Data are presented as the median (IQR) and statistical significance was determined by Kruskal-Wallis test followed by Dunn’s multiple comparison post hoc test. No significant differences were observed among groups at the same time point. IMQ, imiquimod; MTX, methotrexate; NS, normal saline; PASI, psoriasis area and severity index; PLO, pink lotus flower oil.

Representative immunohistochemical staining comparing the expression of total and p-JAK2, -JAK3 and -STAT3 (JAK2/p-JAK2, JAK3/p-JAK3 and STAT3/p-STAT3). Images were captured at x40 magnification (scale bar=50 *μ*m). IMQ, imiquimod; MTX, methotrexate; NS, normal saline; p-, phosphorylated; PLO, pink lotus flower oil.

Histopathological images of major organs, including the (A) liver, (B) kidney and (C) heart, stained with hematoxylin and eosin (scale bar, 200 *μ*m). IMQ, imiquimod; MTX, methotrexate; NS, normal saline; PLO, pink lotus flower oil.

Histopathological images of major organs, including the (A) lung, (B) small intestine and (C) large intestine, stained with hematoxylin and eosin (scale bar, 200 *μ*m). IMQ, imiquimod; MTX, methotrexate; NS, normal saline; PLO, pink lotus flower oil.

## Figures and Tables

**Table I:** Primer sequences.

Gene name	Sequence, 5'-3'	NIH accession no.
*IL-1b*	F: GCAACTGTTCCTGAACTCAACT	AK168047.1
	R: ATCTTTTGGGGTCCGTCAACT	
*IL-6*	F: GACAAAGCCAGAGTCCTTCAGAGAGA	M24221.1
	R: GGTCTTGGTCCTTAGCCACTCCTT	
*K6*	F: GTGGCCTCAGCTCTTCTACC	BC080820.1
	R: TCTGAGCACGGGATTCTGC	
*K16*	F: TGGATGGCGAGAATATCCACAG	AF053235.1
	R: GCTCCTTGAGGATGGACCG	
*K17*	F: GCCCACCTGACTCAGTACAA	BC032161.2
	R: GGAGCTGAGTCCTTAACGGG	
*S100A8*	F: ATCACCATGCCCTCTACAAGAATG	BC078629.1
	R: GTCCAATTCTCTGAACAAGTTTTCG	
*S100A9*	F: CTCTAGGAAGGAAGGACACC	BC027635.1
	R: GCCATCAGCATCATACACTC	
*JAK1*	F: CTCCGAACCGAATCATCACT	NM_146145.2
	R: GCCGTTTTTCTGCTTCTTTG	
*JAK2*	F: TTGTGGTATTACGCCTGTGTATC	L16956.1
	R: ATGCCTGGTTGACTCGTCTAT	
*JAK3*	F: CCATCACGTTAGACTTTGCCA	L40172.1
	R: GGCGGAGAATATAGGTGCCTG	
*STAT1*	F: TCACAGTGGTTCGAGCTTCAG	NM_001205313.1
	R: GCAAACGAGACATCATAGGCA	
*STAT2*	F: TCCTGCCAATGGACGTTCG	NM_019963.2
	R: GTCCCACTGGTTCAGTTGGT	
*STAT3*	F: AGCAGAATCTCAACTTCAGACC	NM_213660.4
	R: TTCGTGGTAAACTGGACACC	
*Nrf2*	F: CAAGACTTGGGCCACTTAAAAGAC	U20532.1
	R: AGTAAGGCTTTCCATCCTCATCAC	
*FLG*	F: TGCTTAAATGCATCTCCAGGT	AF500171.1
	R: TGTTGAAATTTTGAATCTTGGTCCT	
*LOR*	F: GGAGGGGGCTATTACTCCTC	U09189.1
	R: CACCTCCACAGCTACCACCT	
*Cu/Zn-SOD*	F: AACCAGTTGTGTTGTCAGGAC	AH002084.2
	R: CCACCATGTTTCTTAGAGTGAGG	
*Mn-SOD*	F: CAGACCTGCCTTACGACTATGG	NM_013671.3
	R: CTCGGTGGCGTTGAGATTGTT	
*CAT*	F: GGAGGCGGGAACCCAATAG	NM_009804.2
	R: GTGTGCCATCTCGTCAGTGAA	
*GSH-Px*	F: CCACCGTGTATGCCTTCTCC	NM_001329527.1
	R: AGAGAGACGCGACATTCTCAAT	
*β-actin*	F: GGCTGTATTCCCCTCCATCG	NM_007393.5
	R: CCAGTTGGTAACAATGCCATGT	

*CAT*, catalase; F, forward; *FLG*, filaggrin; *GSH-Px*, glutathione peroxidase; *K*, keratin; *LOR*, loricrin; NIH, National Institutes of Health; *Nrf2*, nuclear factor erythroid 2-related factor 2; R, reverse; *SOD*, superoxide dismutase.

## Data Availability

The data generated in the present study may be requested from the corresponding author.
