# Supplementary material for: Pink lotus flower (Nelumbo nucifera) oil extract alleviates imiquimod-induced psoriasis-like dermatitis
Source: Biomed Rep. 2026 Jun 3;25(2):90. doi: 10.3892/br.2026.2163 (PMC13266696; doi:10.3892/br.2026.2163)

Figure S1. PLO improves IMQ-induced psoriatic symptoms in mice. (A) Representative clinical images of dorsal skin on days 1, 3, 5, 7 and 8 following IMQ treatment. PASI scores, including (B) erythema, (C) scaling and (D) skin thickness, and (E) cumulative PASI score were evaluated daily. (F) Body weight of mice in all groups was measured daily (n=10). Data are presented as the median (IQR) and statistical significance was determined by Kruskal-Wallis test followed by Dunn's multiple comparison post hoc test. No significant differences were observed among groups at the same time point. IMQ, imiquimod; MTX, methotrexate; NS, normal saline; PASI, psoriasis area and severity index; PLO, pink lotus flower oil.

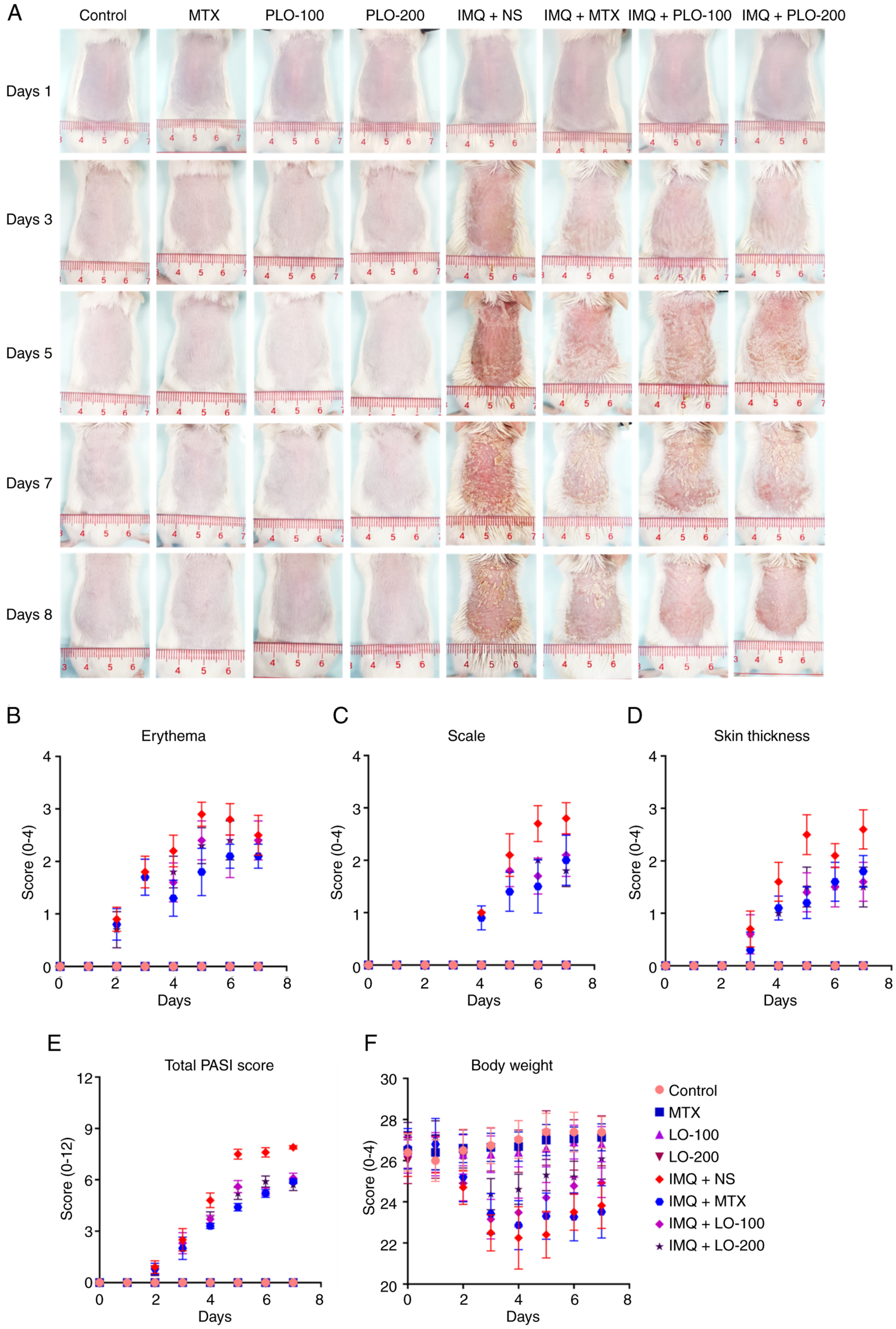

Figure S2. Representative immunohistochemical staining comparing the expression of total and p-JAK2, -JAK3 and -STAT3 (JAK2/p-JAK2, JAK3/p-JAK3 and STAT3/p-STAT3). Images were captured at x40 magnification (scale bar=50 μm). IMQ, imiquimod; MTX, methotrexate; NS, normal saline; p-, phosphorylated; PLO, pink lotus flower oil.

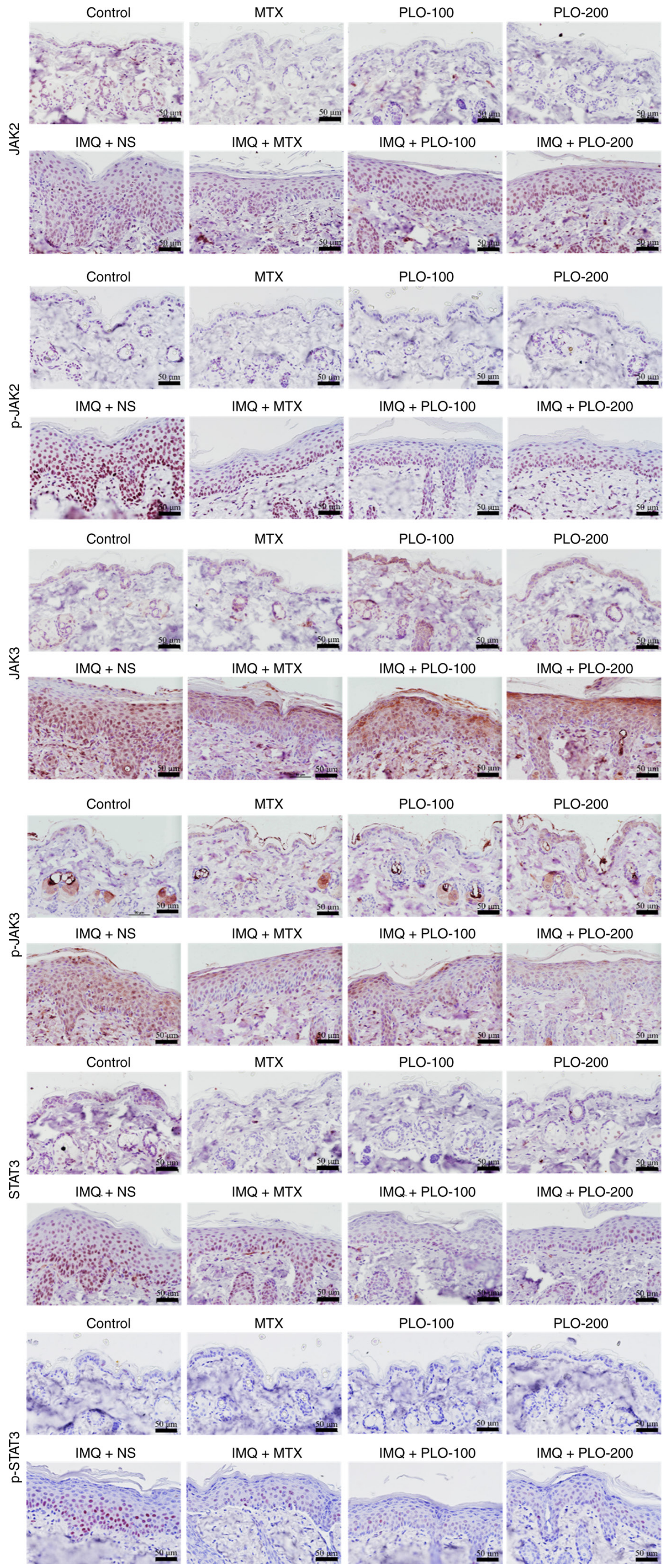

Figure S3. Histopathological images of major organs, including the (A) liver, (B) kidney and (C) heart, stained with hematoxylin and eosin (scale bar, 200 μm). IMQ, imiquimod; MTX, methotrexate; NS, normal saline; PLO, pink lotus flower oil.

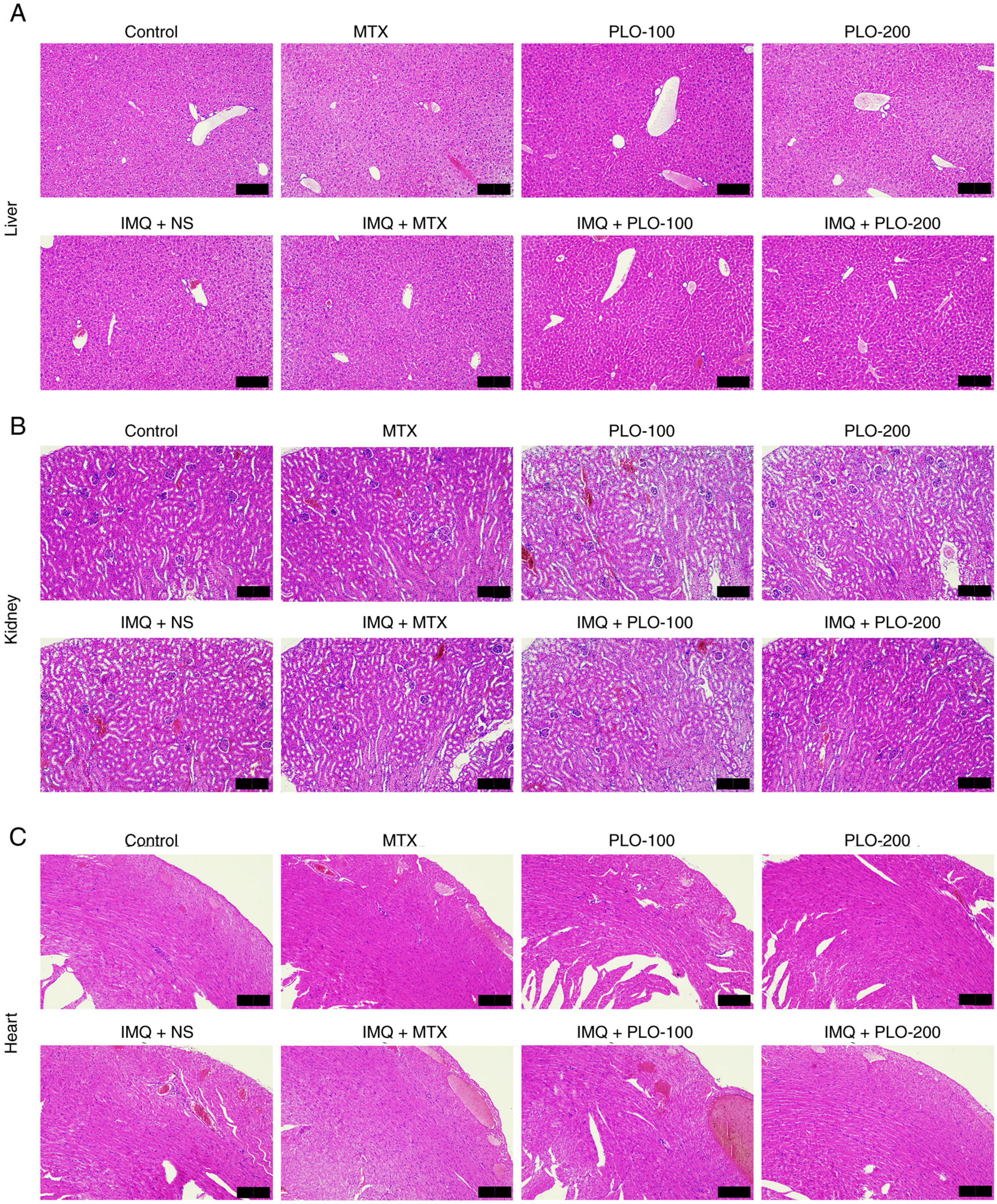

Figure S4. Histopathological images of major organs, including the (A) lung, (B) small intestine and (C) large intestine, stained with hematoxylin and eosin (scale bar, 200 μm). IMQ, imiquimod; MTX, methotrexate; NS, normal saline; PLO, pink lotus flower oil.

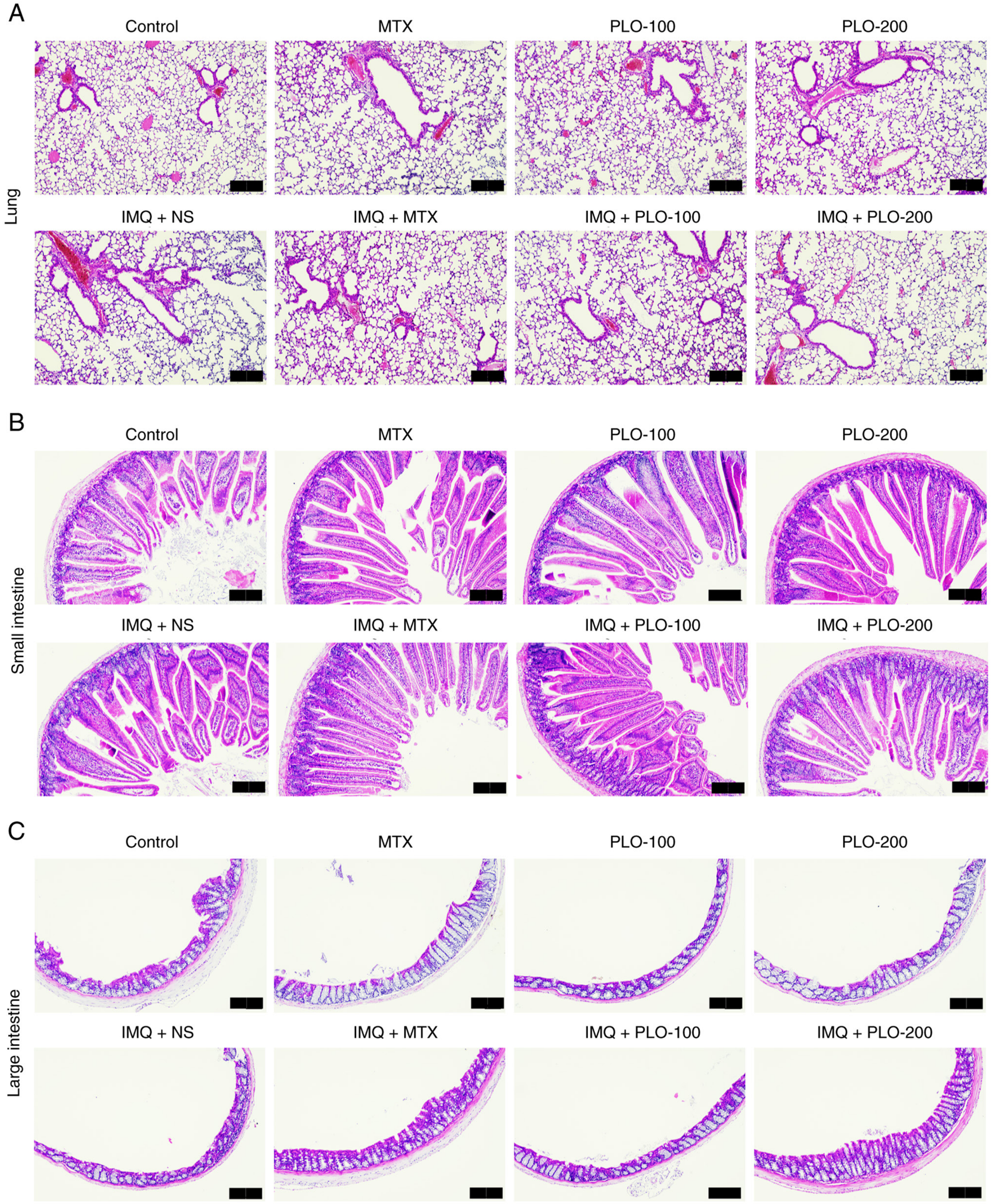

Supplement: PLO improves IMQ-induced psoriatic symptoms in mice. (A) Representative clinical images of dorsal skin on days 1, 3, 5, 7 and 8 following IMQ treatment. PASI scores, including (B) erythema, (C) scaling and (D) skin thickness, and (E) cumulative PASI score were evaluated daily. (F) Body weight of mic [file Supplementary_Data.pdf]
